# Food Safety Knowledge and Foodborne Pathogen Awareness Among Food Truck Customers

**DOI:** 10.3390/foods15111981

**Published:** 2026-06-03

**Authors:** Morooj Farrash, Israa M. Shatwan, Maha A. Althaiban

**Affiliations:** Department of Food and Nutrition, Faculty of Human Sciences and Design, King Abdulaziz University, P.O. Box 80200, Jeddah 21589, Saudi Arabia; mfarrash0006@stu.kau.edu.sa (M.F.); eshatwan@kau.edu.sa (I.M.S.)

**Keywords:** street food safety, hygiene practices, public health risk, consumer awareness, microbial contamination

## Abstract

Food trucks (FTs) are becoming increasingly popular in Saudi Arabia. However, inadequate food safety practices and limited consumer awareness of foodborne pathogens may increase the risk of foodborne illnesses associated with consumption from FTs. Therefore, we assessed consumer knowledge, attitudes, associations with sociodemographic characteristics, and dietary patterns among FT customers. A cross-sectional study was conducted in the Makkah region, Saudi Arabia, between March to October 2025. An online questionnaire was completed by 500 adults covering sociodemographic characteristics, food safety knowledge, attitudes, pathogen awareness, and dietary intake. Data were analyzed using the independent *t*-test, one-way analysis of variance, and linear regression. The mean scores for awareness, knowledge, and attitudes were 16.7 ± 3.8, 14.7 ± 3.3, and 30.8 ± 5.3, respectively. Women had considerably higher knowledge and awareness scores than men (*p* < 0.0001). Significant correlations were found between food safety knowledge and attitude scores (r  =  0.531, *p*  <  0.001), food safety knowledge and awareness scores (r  =  0.633, *p*  <  0.001), and attitude and awareness scores (r  =  0.429, *p*  <  0.001). A trend toward high fruit consumption was observed among participants with high knowledge and awareness scores. These findings highlight the need for targeted consumer education to improve pathogen awareness among FT customers.

## 1. Introduction

Foodborne diseases remain a major public health concern in both developing and developed countries [1,2]. These illnesses are caused by various hazards, including viruses, bacteria, parasites, toxins, metals, and prions [3]. They often arise from inadequate food safety knowledge and/or unsafe food-handling practices among both food handlers and consumers [4]. In 2015, the World Health Organization reported that unsafe food caused approximately 600 million cases of foodborne illness and 420,000 deaths annually [5]. In Saudi Arabia, the Ministry of Health reported 1632 foodborne outbreaks and 11,458 associated illnesses between 2014 and 2018, with nearly half of these outbreaks linked to public food service settings, such as restaurants, catering services, and banquet facilities [6]. As consumers frequently obtain food from restaurants, bakeries, food trucks (FTs), and cafeterias, it is often difficult for them to judge whether proper food-handling practices are being followed. As dining out becomes increasingly common and expectations for hygiene and safety rise, consumers should be aware of the food safety risks associated with these establishments [7]. Such awareness can help individuals make informed choices and may encourage food businesses to maintain high standards of food safety and hygiene.

This context underscores the importance of food safety, which refers to the proper handling, preparation, and storage of food to prevent foodborne diseases [8]. Food safety involves multiple stages throughout the food chain, including the sourcing of raw materials, transportation, storage conditions, personal hygiene of food handlers, and adequate cooking temperatures. Failure at any of these stages can result in microbial, chemical, or physical contamination, potentially leading to serious health consequences and even death [9]. As urban populations expand and food options become more diverse, food safety has emerged as a critical global public health issue affecting individuals across all ages, genders, and income levels. Understanding the risks associated with unsafe food handling therefore helps consumers make informed decisions about where to eat and encourages food establishments to maintain appropriate hygiene and safety standards [7].

Recently, FTs have gained popularity, particularly among adults, because they offer high-quality food in casual and convenient settings [10]. A study conducted in Vietnam reported that consumption of street foods has also been linked to food safety concerns [11]. Consumers often prioritize taste and convenience over safety, thereby increasing their exposure to potential health risks. This growing trend has raised concerns about hygiene and safe food-handling practices, making the prevention of foodborne illness a priority for both consumers and food handlers. Previous reports from countries such as New Zealand, Australia, the United States, Denmark, and India have shown that foodborne disease outbreaks are more common in restaurants and street food settings than in households [12]. Studies from several countries have examined food safety knowledge, attitudes, and awareness among FT or street food customers and have identified gaps in consumer understanding. A study from South Africa reported low food safety knowledge among street food consumers [13]. A study conducted in Poland indicated that consumers perceive a higher risk when consuming food from street vendors compared to other food service establishments, reflecting concerns about potential contamination in informal food settings [14]. Studies from Bangladesh and Portugal found moderate knowledge levels but limited awareness of specific foodborne pathogens among street food patrons [2,5]. In Saudi Arabia, moderate food safety knowledge and positive attitudes have been reported among customers of FTs [15].

Despite previous research, gaps remain in our understanding of food safety knowledge, attitudes, awareness of foodborne pathogens, and consumption patterns among FT customers in Saudi Arabia. Thus, in the current study, we evaluated these aspects among adult FT customers in the Makkah region, Saudi Arabia. The associations between consumers’ sociodemographic characteristics, household income levels, food safety knowledge, attitudes, awareness, and consumption patterns from FTs were examined because these factors influence individuals’ access to health information and food safety practices, which may help to improve consumer food safety awareness and promote healthier dietary behaviors. This study’s results are intended to support the development of targeted food safety education and public health interventions to reduce foodborne disease risks associated with consumption from FTs and improve consumer health outcomes.

We hypothesized that sociodemographic factors of FT customers would be significantly associated with their knowledge and attitude about food safety as well as foodborne pathogen awareness. Additionally, the knowledge, attitude, and awareness of FT customers would be significantly associated with their food consumption. The main key findings are the effect of sociodemographic factors (gender, age, education, and income) on knowledge and attitude about food safety and foodborne pathogen awareness, and whether high knowledge, attitude, and awareness are associated with high consumption of healthy food groups and low from unhealthy ones. The findings provide evidence to inform targeted educational and policy interventions for improving food safety knowledge, awareness and attitude among food truck customers.

## 2. Materials and Methods

### 2.1. Study Design

This was a cross-sectional study designed with a quantitative approach to assess food safety knowledge, attitudes, awareness of foodborne pathogens, and consumption patterns among FT customers.

### 2.2. Sampling Procedures

According to the General Authority for Statistics, the population of the Makkah region during the data collection period was 5,376,462 individuals aged ≥ 18 years, including both men and women [16]. Based on a 95% confidence level, 5% margin of error, and 50% response distribution, the required sample size was calculated to be 500 participants to ensure adequate statistical power [17]. The inclusion criteria for individuals were age ≥ 18 years, residency in the Makkah region, and having consumed food from FTs. A convenience sample method was used to sample from three main urban cities in the Makkah region (Jeddah, Makkah, and Taif) between March to October 2025. This sampling method is considered fast, cost-effective, and practical to reach available participants but may introduce sampling bias [18].

### 2.3. Data Collection

Data were collected using a structured questionnaire adapted from previous studies [15,19,20]. The questionnaire was translated into Arabic, converted into an online format, and distributed via email, WhatsApp, X, and Telegram. In addition, the survey link was converted into a barcode, printed, and displayed beside selected FTs in the Makkah region. To ensure content validity, the questionnaire was reviewed by experts in the department. A pilot study involving 15 participants was conducted to assess clarity before full distribution. Internal consistency reliability was evaluated using Cronbach’s alpha, yielding values of 0.775 for the knowledge scale, 0.695 for the attitude scale, and 0.761 for the awareness scale, indicating acceptable reliability.

The questionnaire consisted of five sections. The first section collected sociodemographic information, including age, gender, educational level, income level, marital status, and occupation. The second and third sections were adapted from a previous study by Madilo et al. [19].

The second section included 19 questions assessing consumers’ food safety knowledge. The third section included 25 questions evaluating awareness of foodborne pathogens, including knowledge of symptoms and methods of preventing foodborne illness. In both sections, response options were “yes,” “no,” and “do not know.” For scoring, correct answers were coded as 1, whereas incorrect and “do not know” responses were coded as 0. The sum of question scores in each section was then calculated for each participant to provide the total knowledge and awareness score [19]. Based on the median score, knowledge scores < 15 were classified as low knowledge, whereas scores ≥ 15 were classified as high knowledge. Similarly, awareness scores < 17 were categorized as low awareness, whereas scores ≥ 17 were categorized as high awareness. Knowledge and awareness studies frequently stratify data based on median because it helps with comparisons between groups as well as identifying critical knowledge and awareness gaps within a population [20,21].

The fourth section was adapted from a previous study by Alhashim et al. [15] and included 13 items assessing FT customers’ attitudes toward food safety, including food and personal hygiene, cross-contamination, food handler health status, and cleaning practices. Response options were “agree,” “disagree,” “neutral,” and “do not know,” scored on a 4-point scale as follows: agree = 3, neutral = 2, disagree = 1, and do not know = 0. A composite mean attitude score ranging from 1 to 3 was calculated for each participant, with higher scores indicating a more positive attitude. Scores < 2 were categorized as negative attitudes, scores from 2.0 to 2.5 as neutral attitudes, and scores from 2.6 to 3.0 as positive attitudes [15].

The fifth section was adapted from a previous study by West et al. [22] and consisted of 15 food frequency questionnaire (FFQ) items assessing dietary intake among FT customers. This section covered key dietary components, including fruit, vegetables, meat, sugar-sweetened beverages, salty snacks, and sweets. Response options were “never/rarely,” “less than once a week,” “once a week,” “2–3 times a week,” and “4–6 times a week” and were coded as 0, 1, 2, 3, and 4, respectively.

### 2.4. Statistical Analysis

Statistical analyses were performed using SPSS software version 26. Data are presented as frequencies and percentages for categorical variables and as mean ± standard deviation for knowledge, attitude, and awareness scores. The normality of each score variable was assessed using the Kolmogorov–Smirnov test. Data not normally distributed underwent Log10 transformations. The Chi-square test was used to examine associations between sociodemographic variables, including age, gender, educational level, marital status, occupation, and household income (<5000 Saudi Riyals [SR], 5000–15,000 SR, and >15,000 SR), and knowledge, attitude, and awareness categories. The independent *t*-test and one-way analysis of variance were used to assess associations between sociodemographic and FFQ variables and knowledge, attitude, and awareness scores. In addition, linear regression analysis was performed to examine the associations of sociodemographic variables with knowledge, attitude, and awareness scores. The confounders included in the model were age, gender, educational level, income level, marital status, and occupation, depending on the factor being tested.

Multiple testing correction using the Bonferroni method was applied to the tested hypotheses (first hypothesis ’’sociodemographic factors of FT customers would be significantly associated with their knowledge and attitude about food safety as well as foodborne pathogen awareness’’ 0.05/6 sociodemographic factors × 3 knowledge, attitude, and awareness and the adjusted *p*-value was 0.002; second hypothesis ‘’ the knowledge, attitude, and awareness of FT customers would be significantly associated with their food consumption’’ 0.05/15 food groups × 3 knowledge, attitude, and awareness and the adjusted *p*-value was 0.001).

## 3. Results

Participant characteristics are presented in Table 1. Most participants were women (75.8%), and 30.6% were >45 years. More than half held a bachelor’s degree (59.4%), whereas 21.8% had a postgraduate degree and 18.8% had a high school education or lower. Regarding monthly household income, 43.8% reported earning <5000 SR, 35.8% reported earning 5000–15,000 SR, and 20.4% reported earning >15,000 SR. Participants were almost evenly split by marital status, with 51.4% unmarried and 48.6% married. In terms of occupation, 31.2% were students, 31.0% were employed, 21.0% were unemployed, and 16.8% were retired. The mean awareness, knowledge, and attitude scores were 16.7 ± 3.8, 14.7 ± 3.3, and 30.8 ± 5.3, respectively.

Table 2 presents the associations of sociodemographic characteristics with food safety knowledge, attitude, and awareness scores among FT customers. Only the association between knowledge and awareness scores and gender remained significant (*p* < 0.0001), after adjustment and Bonferroni correction. Attitude score was not significantly associated with any sociodemographic factors.

Table 3, Table 4 and Table 5 summarize the associations between sociodemographic characteristics and categorized levels of food safety knowledge, attitudes, and pathogen awareness among FT customers. As shown in Table 3, knowledge level differed considerably by gender. Among men, 60.3% were classified as having low knowledge, whereas 52.2% of women were classified as having high knowledge. Educational level also showed an association with knowledge level: 56.0% of participants with high knowledge held a postgraduate degree, whereas 64.9% of those with low knowledge had a high school education or lower. No significant associations were found between knowledge level and sociodemographic factors. Table 4 presents the distribution of negative, neutral, and positive attitude levels across sociodemographic categories. No significant differences in attitude level were found according to gender, age, educational level, income, marital status, or occupation (*p* > 0.05). Table 5 shows that gender was significantly associated with awareness level of foodborne pathogens. A higher proportion of men (62.8%) was in the low-awareness group, whereas 57.5% of women were classified in the high-awareness group. No other significant associations were observed with awareness level.

Table 6, Table 7 and Table 8 presents the associations between food safety knowledge, attitudes, awareness of foodborne pathogens, and FFQ responses among FT customers. Only fruit consumption showed an association with knowledge level, where participants with higher knowledge scores reported greater fruit consumption compared with those with lower knowledge scores (*p* = 0.023; Table 6). No significant differences were observed between most food groups and food safety attitudes (Table 7). Concerning awareness of foodborne pathogens, fruit consumption was the only food group associated with awareness level, with participants who had higher awareness reporting greater fruit consumption than others (*p* = 0.037; Table 8).

Table 9 summarizes a strong positive association between food safety knowledge, attitudes, and awareness of foodborne pathogens scores among FT customers (*p* < 0.001). However, the correlation between knowledge, attitudes, and awareness was moderate to weak.

## 4. Discussion

In the present study, we evaluated the food safety knowledge, attitudes, and awareness of foodborne pathogens of FT customers in the Makkah region of Saudia Arabia and assessed their association with sociodemographic characteristics and dietary consumption patterns. The findings showed that FT customers in the three largest cities in the Makkah region had good levels of food safety knowledge, attitudes, and awareness of foodborne pathogens. Gender was associated with knowledge and awareness, with women having higher levels. Knowledge, attitude, and awareness scores were significantly correlated. There was a trend toward high fruit intake among FT customers with a high level of knowledge, but no significant association was observed between knowledge, attitude, and awareness and food intake.

We found that FT customers’ food safety knowledge ranged from moderate to high, with most participants demonstrating a solid understanding of basic food safety concepts, including foodborne diseases, risks associated with raw and undercooked foods, hygiene, and prevention of cross-contamination. In addition, they showed high level of awareness regarding food allergens, expired foods, and chemical contaminants, such as insecticides, as potential food safety hazards. This finding is consistent those of previous studies conducted in Bangladesh [5] and Portugal [2], which reported moderate levels of knowledge alongside notable misconceptions regarding food safety, foodborne diseases, and microbial hazards. Similarly, an integrative review conducted in Gulf countries (Saudi Arabia, Qatar, UAE, Bahrain, Oman, and Kuwait) reported that consumers generally demonstrated acceptable knowledge regarding several food safety concepts, particularly those related to temperature control during food storage, safe food storage practices, prevention of cross-contamination, and the safe handling of raw or undercooked foods. However, the review also identified persistent gaps in some food safety practices among consumers [23]. In contrast, a study from South Africa [13] found that street food consumers reported low confidence in their food safety knowledge. These differences may reflect variations in socioeconomic conditions, general education levels, regulatory frameworks governing street food operations, and the intensity of national food safety campaigns.

In the present study, higher knowledge scores were strongly associated with women, which agrees with a previous study from Saudi Arabia [24] but not with studies conducted in the UAE [25], Egypt [26], and Jordon [27] where women had low food safety knowledge. The differences could be due to educational levels and low initiatives aimed toward women among these countries. The association between gender and knowledge is often explained by women’s greater involvement in food preparation and household food decisions [28,29]. Collectively, the overall level of knowledge among FT customers in the Makkah region is encouraging.

We found that food safety attitudes among FT customers were generally positive. This result agrees with a study conducted in Vietnam [11], which reported that consumers demonstrated a positive attitude toward food hygiene and safety standards among food handlers. They particularly emphasized the importance of personal hygiene practices, including hand cleanliness, proper clothing, and the overall cleanliness of food preparation areas when evaluating food facilities. Similarly, a previous study conducted in the Asir region of Saudi Arabia among customers of FTs operated by productive families reported favorable food safety attitudes [15].

Our findings revealed a limited awareness of foodborne pathogens among consumers, in contrast to their relatively stronger knowledge and attitudes. A considerable proportion of participants reported being unaware of major pathogens including, *Escherichia coli, Campylobacter jejuni, Listeria monocytogenes*, and *Staphylococcus aureus*. This level of awareness was lower than that reported in a study conducted in Ghana, where awareness of foodborne pathogens was relatively high [30]. This discrepancy may be partly explained by differences in the study populations. The present study included a broad sample of consumers from the general community. Conversely, the Ghanaian study focused specifically on university students, who are more likely to encounter pathogen-specific information through academic coursework and campus-based awareness programs. Several misconceptions were identified in the present study: some participants believed that washing vegetables in salt solution or simply refrigerating food is sufficient to eliminate pathogens, and many incorrectly identified headache as a typical symptom of foodborne illness. Such misunderstandings may increase the risk of foodborne disease, particularly in the FT setting, where time–temperature control and prevention of cross-contamination are critical [31]. These misconceptions further suggest that many consumers mainly associate foodborne illness with obvious gastrointestinal symptoms and may not recognize that nonspecific symptoms can also occur, potentially delaying recognition and appropriate response. Addressing these gaps requires focused educational messages that clearly explain both the limitations of common household practices and range of symptoms associated with foodborne illness.

We found that female FT customers had considerably higher awareness scores than men, which is consistent with previous studies from Saudi Arabia, Portugal, and Bangladesh [2,5,21]. This could be explained by the traditional role of women as primary food preparers in many households, which may increase their exposure to food safety information and strengthen their motivation to protect family health. These findings underscore the importance of tailoring food safety interventions to specific demographic groups, particularly men, using communication channels and messages suited to each group.

We also found a trend toward high fruit intake among FT customers with high knowledge and awareness levels suggesting that better-informed individuals may be more likely to adopt certain healthier dietary choices. Although relatively few studies have explicitly combined food safety knowledge measures with FFQ-based dietary assessments, the present findings are consistent with previous research which showed that higher food safety-related knowledge was modestly associated with healthier dietary patterns, particularly higher intakes of fruits, vegetables, and other core food groups aligned with dietary guidelines [32]. Thus, positive cognition may be related to health, and food safety can influence some aspects of dietary behavior, although the observed associations are generally modest. Nevertheless, the absence of consistent associations across all food groups and awareness levels indicates that improvements in knowledge and attitudes alone are unlikely to be sufficient to shift overall dietary patterns toward optimal health. Comprehensive strategies that combine education with environmental changes, such as healthier FT menus and clearer point-of-purchase information, together with economic incentives, are therefore likely needed to achieve meaningful and sustainable behavior change among FT customers [33,34,35].

The correlation result indicated moderate to strong association between knowledge, attitude, and awareness. However, weak correlation was observed between attitude and awareness. Similarly, previous studies reported similar findings between knowledge and attitude among Malaysian FT handlers [36], Ethiopian food handlers [37], and Bangladeshi students [38].

This study has several strengths. It focused on an understudied consumer group and provided context-specific insights from a setting wherein street food is becoming increasingly popular. The relatively large sample size and inclusion of participants from diverse sociodemographic backgrounds, including different age, education, income, and occupation groups, enabled meaningful subgroup analyses and identification of vulnerable groups with lower knowledge or awareness. The use of adapted questionnaires and appropriate statistical analyses, including adjusted models, further strengthens the internal validity of the findings.

Some limitations should be considered. First, the cross-sectional design does not allow causal relationships to be established between sociodemographic characteristics, knowledge, attitudes, awareness, and dietary behaviors; only associations can be inferred. Second, the data were based on self-reported questionnaires and are therefore subject to recall and social desirability bias, which may have led participants to overestimate their knowledge or report healthier behaviors than they actually practice. Furthermore, participant recruitment through online platforms may have introduced selection bias by favoring younger individuals, those who are more technologically engaged, or participants with greater interest in food safety and health-related issues, which may have influenced the overall level of reported knowledge and attitudes. The sample was also predominantly composed of women, which may limit the representativeness of the study population and reduce result generalizability to the broader population of FT customers, particularly male customers. The skewedness of the sample toward women may be because women tend to have the dominant decision about purchasing products.

Despite these limitations, the study provides valuable evidence that can inform targeted educational and policy interventions aimed at improving food safety among FT customers.

## 5. Conclusions

This study is one of the few studies assessing the FT customers food safety knowledge, attitudes, and awareness of foodborne pathogens in Saudi Arabia. Women had significantly higher scores of knowledge and awareness compared with men. Thus, a strong positive association was conclusively observed between food safety knowledge, attitudes, and awareness of foodborne pathogens scores among FT customers. Our findings highlight the importance of planning national strategies for reducing foodborne disease risks and improving public health knowledge and awareness. Future studies should adopt longitudinal designs and incorporate microbial analyses of FT food samples to better examine the relationship between consumption patterns and disease occurrence. In addition, extending this research to other regions of Saudi Arabia would help determine result generalizability and support the development of standardized national food safety regulations for FT establishments.

## Figures and Tables

**Table 1:** Frequencies and percentages of the sociodemographic characteristics of FT customers, along with awareness, knowledge, and attitude scores.

Characterization	Total (n = 500) n (%)
**Gender**	
Men	121 (24.2%)
Women	379 (75.8%)
**Age**	
18–25	141(28.2%)
26–35	130 (26.0%)
35–45	76 (15.2%)
>45	153 (30.6%)
**Educational level**	
High school or below	94 (18.8%)
Bachelor	297 (59.4%)
Postgraduate	109 (21.8%)
**Income level**	
<5000 SR	219 (43.8%)
5000–15,000 SR	179 (35.8%)
>15,000 SR	102 (20.4%)
**Marital status**	
Married	243 (48.6%)
Unmarried	257 (51.4%)
**Occupation**	
Student	156 (31.2%)
Employed	155 (31.0%)
Unemployed	105 (21.0%)
Others, such as retired	84 (16.8%)
**Scores**	**Mean ± SD**
Awareness	16.7 ± 3.8
Knowledge	14.7 ± 3.3
Attitudes	30.8 ± 5.3

Awareness, knowledge, and attitude scores are presented as mean ± SD. FT, food truck; SD, standard deviation.

**Table 2:** Association of food safety knowledge, attitude, and awareness of foodborne pathogen scores with the sociodemographic characteristics of FT customers.

Sociodemographic Characteristics	Knowledge	Attitude	Awareness
	Mean ± SD	Mean ± SD	Mean ± SD
**Gender ** ^++^			
Men	13.6 ± 4.0	29.9 ± 6.2	15.4 ± 4.2
Women	15.1 ± 2.8	31.1 ± 4.9	17.1 ± 3.6
***p*****-value**	0.004	0.004	0.005
**Adjusted *****p*****-value**	<0.0001 *	0.461	<0.0001 *
**Age ** ^+^			
18–25	14.1 ± 3.5	29.8 ± 6.6	16.6 ± 3.9
26–35	15.3 ± 2.7	31.8 ± 3.5	17.4 ± 3.7
35–45	14.6 ± 3.2	30.6 ± 4.9	16.3 ± 3.6
>45	14.8 ± 3.5	30.9 ± 5.1	16.4 ± 3.9
***p*****-value**	0.087	0.098	0.173
**Adjusted *****p*****-value**	0.216	0.224	0.512
**Educational level ** ^+^			
High school or below	14.0 ± 3.4	30.9 ± 4.5	16.0 ± 3.4
Bachelor	14.8 ± 3.3	30.7 ± 5.6	16.7 ± 3.9
Postgraduate	15.2 ± 3.2	31.1 ± 4.9	17.6 ± 3.7
***p*****-value**	0.013	0.614	0.032
**Adjusted *****p*****-value**	0.170	0.434	0.234
**Income level**			
<5000 SR	14.5 ± 3.4	30.9 ± 5.2	16.7 ± 3.8
5000–15,000 SR	14.9 ± 2.8	31.3 ± 3.9	16.8 ± 3.8
>15,000 SR	14.79 ± 3.9	29.6 ± 6.8	16.7 ± 4.1
***p*****-value**	0.423	0.082	0.864
**Adjusted *****p*****-value**	0.399	0.051	0.587
**Marital status** ^++^			
Married	14.8 ± 3.5	30.8 ± 5.3	16.6 ± 3.8
Unmarried	14.6 ± 3.1	30.8 ± 5.2	16.8 ± 3.8
***p*****-value**	0.006	0.005	0.006 *
**Adjusted *****p*****-value**	0.825	0.793	0.942
**Occupation** ^+^			
Student	14.6 ± 3.3	30.5 ± 5.9	16.9 ± 3.9
Employed	15.2 ± 3.1	30.9 ± 5.3	16.8 ± 3.6
Unemployed	14.2 ± 3.5	30.8 ± 5.3	16.5 ± 3.7
Other (e.g., retired)	14.9 ± 3.3	31.3 ± 3.7	16.5 ± 4.1
***p*****-value**	0.143	0.817	0.600
**Adjusted *****p*****-value**	0.050	0.583	0.581

^+^ One-way analysis of variance and ^++^ independent-samples *t*-test were used to examine group differences. Adjusted *p*-values were obtained using regression analysis adjusted for gender, age, educational level, marital status, and occupation, depending on the factor being tested. * *p* < 0.001.

**Table 3:** Association between food safety knowledge level (high vs. low) and the sociodemographic characteristics of respondents.

Knowledge Regarding Safety of Food Sold by FTs			
Sociodemographic Characteristics	High Knowledge	Low Knowledge	*p*-Value
**Gender**			
Men	48 (39.7%)	73 (60.3%)	0.016 *
Women	198 (52.2%)	181 (47.8%)	
**Age**			
18–25	58 (41.1%)	83 (58.9%)	0.152
26–35	70 (53.8%)	60 (46.2%)	
35–45	39 (51.3%)	37 (48.7%)	
>45	79 (51.6%)	74 (48.4%)	
**Educational level**			
High school or below	33 (35.1%)	61 (64.9%)	0.007 *
Bachelor	152 (51.2%)	145 (48.8%)	
Postgraduate	61 (56.0%)	48 (44.0%)	
**Income level**			
<5000 SR	101 (46.1%)	118 (53.9%)	0.337
5000–15,000 SR	89 (49.7%)	90 (50.3%)	
>15,000 SR	56 (54.9%)	46 (45.1%)	
**Marital status**			
Married	126 (51.9%)	117 (48.1%)	0.249
Unmarried	120 (46.7%)	137 (53.3%)	
**Occupation**			
Student	73 (46.8%)	83 (53.2%)	0.104
Employed	88 (56.8%)	67 (43.2%)	
Unemployed	44 (41.9%)	61 (58.1%)	
Other (e.g., retired)	41 (48.8%)	43 (51.2%)	

The Chi-square test was used to examine differences between knowledge levels. * *p* < 0.05.

**Table 4:** Association between food safety attitude level (positive, neutral, or negative) and the sociodemographic characteristics of respondents.

Attitude Regarding Safety of Food Sold by FTs				
Sociodemographic Characteristics	Positive	Neutral	Negative	*p*-Value
**Gender**				
Men	45 (30.2%)	58 (47.9%)	18 (14.9%)	0.071
Women	168 (44.3%)	180 (47.5%)	31 (8.2%)	
**Age**				
18–25	51 (36.2%)	72 (51.1%)	18 (12.8%)	0.219
26–35	60 (46.2%)	64 (49.2%)	6 (4.6%)	
35–45	32 (42.1%)	35 (46.1%)	9 (11.8%)	
>45	70 (45.8%)	67 (43.8%)	16 (10.5%)	
**Educational level**				
High school or below	31 (33.0%)	54 (57.4%)	9 (9.6%)	0.142
Bachelor	132 (44.4%)	132 (44.4%)	33 (11.1%)	
Postgraduate	50 (45.9%)	52 (47.7%)	7 (6.4%)	
**Income level**				
<5000 SR	91 (41.6%)	111 (50.7%)	17 (7.8%)	0.319
5000–15,000 SR	83 (46.4%)	78 (43.6%)	18 (10.1%)	
>15,000 SR	39 (38.2%)	49 (48.0%)	14 (13.7%)	
**Marital status**				
Married	101 (41.6%)	118 (48.6%)	24 (9.9%)	0.899
Unmarried	112 (43.6%)	120 (46.7%)	25 (9.7%)	
**Occupation**				
Student	62 (39.7%)	79 (50.6%)	15 (9.6%)	0.906
Employed	68 (43.9%)	73 (47.1%)	14 (9.0%)	
Unemployed	44 (41.9%)	48 (45.7%)	13 (12.4%)	
Other (e.g., retired)	39 (46.4%)	38 (45.2%)	7 (8.3%)	

The Chi-square test was used to examine differences across attitude levels.

**Table 5:** Association between foodborne pathogen awareness level (high vs. low) and the sociodemographic characteristics of respondents.

Awareness of Foodborne Pathogens Associated with Food Sold by FTs			
Sociodemographic Characteristics	High Awareness	Low Awareness	*p*-Value
**Gender**			
Men	45 (37.2%)	76 (62.8%)	<0.0001 *
Women	218 (57.5%)	161 (42.5%)	
**Age**			
18–25	70 (49.6%)	71 (50.4%)	0.046
26–35	80 (61.5%)	50 (38.5%)	
35–45	32 (42.1%)	44 (57.9%)	
>45	81 (52.9%)	72 (47.1%)	
**Educational level**			
High school or below	41 (43.6%)	53 (56.4%)	0.010
Bachelor	152 (51.2%)	145 (48.8%)	
Postgraduate	70 (64.2%)	39 (35.8%)	
**Income level**			
<5000 SR	112 (51.1%)	107 (48.9%)	0.765
5000–15,000 SR	98 (54.7%)	81 (45.3%)	
>15,000 SR	53 (52.0%)	49 (48.0%)	
**Marital status**			
Married	125 (51.4%)	118 (48.6%)	0.614
Unmarried	138 (53.7%)	119 (46.3%)	
**Occupation**			
Student	82 (52.6%)	74 (47.4%)	0.894
Employed	83 (53.5%)	72 (46.5%)	
Unemployed	52 (49.5%)	53 (50.5%)	
Other (e.g., retired)	46 (54.8%)	38 (45.2%)	

The Chi-square test was used to examine differences between awareness levels. * *p* < 0.0001.

**Table 6:** Association between food frequency questionnaire responses and food safety knowledge among FT customers.

	High Knowledge	Low Knowledge	
Food Type	Mean ± SD	Mean ± SD	*p*-Value
Fruits	2.36 ± 1.074	1.93 ± 1.088	0.023 *
Vegetables	2.74 ± 1.049	2.43 ± 1.186	0.212
Dairy products	2.46 ± 1.159	2.26 ± 1.165	0.340
Beans and legumes	1.78 ± 1.028	1.48 ± 1.062	0.768
Breakfast cereals	1.20 ± 1.169	0.91 ± 1.018	0.560
Whole-grain bread	2.19 ± 1.349	1.98 ± 1.363	0.561
White meat (poultry)	2.93 ± 0.973	2.83 ± 1.013	0.635
Red meat	2.34 ± 1.040	1.99 ± 1.216	0.276
Fish	1.56 ± 1.126	1.47 ± 1.058	0.517
Sugar-sweetened beverages/sodas	1.68 ± 1.375	1.75 ± 1.342	0.913
Potato chips or salty snacks	1.70 ± 1.208	1.77 ± 1.227	0.465
Sweets (e.g., chocolate, cake, donuts, cookies)	2.13 ± 1.222	2.05 ± 1.221	0.581
Pastries, pies, or sausage rolls	1.80 ± 1.126	1.74 ± 1.213	0.625
Fast food (e.g., McDonald’s, KFC)	1.39 ± 1.169	1.43 ± 1.229	0.839
Pizza (store-bought or homemade)	1.47 ± 1.068	1.40 ± 1.073	0.606

The independent-samples *t*-test was used to examine differences between knowledge levels. * *p* < 0.05 was considered significant.

**Table 7:** Association between food frequency questionnaire responses and food safety attitudes among FT customers.

	Positive Attitudes	Neutral Attitudes	Negative Attitudes	
Food Type	Mean ± SD	Mean ± SD	Mean ± SD	*p*-Value
Fruits	2.28 ± 1.052	2.06 ± 1.164	1.92 ± 0.932	0.305
Vegetables	2.64 ± 1.088	2.58 ± 1.155	2.31 ± 1.176	0.330
Dairy products	2.41 ± 1.208	2.39 ± 1.112	2.00 ± 1.190	0.404
Beans and legumes	1.72 ± 1.039	1.58 ± 1.068	1.47 ± 1.043	0.631
Breakfast cereals	1.25 ± 1.161	0.83 ± 0.976	1.29 ± 1.225	0.140
Whole-grain bread	2.18 ± 1.316	2.05 ± 1.376	1.88 ± 1.452	0.859
White meat (poultry)	2.82 ± 1.064	2.98 ± 0.893	2.67 ± 1.107	0.184
Red meat	2.17 ± 1.150	2.16 ± 1.129	2.12 ± 1.218	0.141
Fish	1.55 ± 1.117	1.47 ± 1.074	1.57 ± 1.080	0.179
Sugar-sweetened beverages/sodas	1.66 ± 1.339	1.73 ± 1.340	1.92 ± 1.525	0.263
Potato chips or salty snacks	1.69 ± 1.204	1.77 ± 1.212	1.80 ± 1.307	0.211
Sweets (e.g., chocolate, cake, donuts, cookies)	1.96 ± 1.201	2.21 ± 1.192	2.06 ± 1.405	0.473
Pastries, pies, or sausage rolls	1.75 ± 1.116	1.79 ± 1.185	1.78 ± 1.343	0.525
Fast food (e.g., McDonald’s, KFC)	1.31 ± 1.133	1.45 ± 1.234	1.61 ± 1.288	0.704
Pizza (store-bought or homemade)	1.44 ± 1.065	1.42 ± 1.051	1.47 ± 1.192	0.855

A one-way analysis of variance was used to examine differences across attitude levels.

**Table 8:** Association between food frequency questionnaire responses and awareness of foodborne pathogens among FT customers.

	High Awareness	Low Awareness	
Food Type	Mean ± SD	Mean ± SD	*p*-Value
Fruits	2.32 ± 1.100	1.94 ± 1.070	0.037 *
Vegetables	2.73 ± 1.112	2.41 ± 1.130	0.094
Dairy products	2.46 ± 1.168	2.25 ± 1.155	0.254
Beans and legumes	1.70 ± 1.068	1.54 ± 1.035	0.823
Breakfast cereals	1.16 ± 1.152	0.93 ± 1.033	0.101
Whole-grain bread	2.21 ± 1.349	1.95 ± 1.360	0.769
White meat (poultry)	2.92 ± 1.012	2.84 ± 0.974	0.475
Red meat	2.27 ± 1.099	2.04 ± 1.184	0.474
Fish	1.61 ± 1.126	1.41 ± 1.044	0.640
Sugar-sweetened beverages/sodas	1.70 ± 1.375	1.73 ± 1.341	0.101
Potato chips or salty snacks	1.76 ± 1.204	1.71 ± 1.232	0.954
Sweets (e.g., chocolate, cake, donuts, cookies)	2.14 ± 1.213	2.03 ± 1.230	0.498
Pastries, pies, or sausage rolls	1.84 ± 1.152	1.70 ± 1.188	0.669
Fast food (e.g., McDonald’s, KFC)	1.42 ± 1.166	1.40 ± 1.237	0.704
Pizza (store-bought or homemade)	1.46 ± 1.051	1.41 ± 1.092	0.284

The independent-samples *t*-test was used to examine differences between awareness levels. * *p* < 0.05 was considered significant.

**Table 9:** Association between food safety knowledge, attitude, and awareness of foodborne pathogens among FT customers.

Variable		Knowledge	Attitudes	Awareness
**Knowledge**	Person correlation coefficient (r)	1	0.531 ****	0.633 ****
	Sig (2-Tailed)		<0.001	<0.001
**Attitudes**	Person correlation coefficient (r)	0.531 ****	1	0.392 ****
	Sig (2-Tailed)	<0.001		<0.001
**Awareness**	Person correlation coefficient (r)	0.633 ****	0.392 ****	1
	Sig (2-Tailed)	<0.001	<0.001	

**** *p* < 0.001.

## Data Availability

The original contributions presented in the study are included in the article, further inquiries can be directed to the corresponding author.

## Abbreviations

The following abbreviations are used in this manuscript:

FFQ	food frequency questionnaire
FTs	food trucks
SD	standard deviation
SPSS	Statistical Package for the Social Sciences
SR	Saudi Riyal
